# Detoxification of Multiple Heavy Metals by a Half-Molecule ABC Transporter, HMT-1, and Coelomocytes of *Caenorhabditis elegans*

**DOI:** 10.1371/journal.pone.0009564

**Published:** 2010-03-05

**Authors:** Marc S. Schwartz, Joseph L. Benci, Devarshi S. Selote, Anuj K. Sharma, Andy G. Y. Chen, Hope Dang, Hanna Fares, Olena K. Vatamaniuk

**Affiliations:** 1 Department of Crop and Soil Sciences, Cornell University, Ithaca, New York, United States of America; 2 Department of Molecular and Cellular Biology, University of Arizona, Tucson, Arizona, United States of America; Brown University, United States of America

## Abstract

**Background:**

Developing methods for protecting organisms in metal-polluted environments is contingent upon our understanding of cellular detoxification mechanisms. In this regard, half-molecule ATP-binding cassette (ABC) transporters of the HMT-1 subfamily are required for cadmium (Cd) detoxification. HMTs have conserved structural architecture that distinguishes them from other ABC transporters and allows the identification of homologs in genomes of different species including humans. We recently discovered that HMT-1 from the simple, unicellular organism, *Schizosaccharomyces pombe*, SpHMT1, acts independently of phytochelatin synthase (PCS) and detoxifies Cd, but not other heavy metals. Whether HMTs from multicellular organisms confer tolerance only to Cd or also to other heavy metals is not known.

**Methodology/Principal Findings:**

Using molecular genetics approaches and functional *in vivo* assays we showed that HMT-1 from a multicellular organism, *Caenorhabditis elegans*, functions distinctly from its *S. pombe* counterpart in that in addition to Cd it confers tolerance to arsenic (As) and copper (Cu) while acting independently of *pcs-1*. Further investigation of *hmt-1* and *pcs-1* revealed that these genes are expressed in different cell types, supporting the notion that *hmt-1* and *pcs-1* operate in distinct detoxification pathways. Interestingly, *pcs-1* and *hmt-1* are co-expressed in highly endocytic *C. elegans* cells with unknown function, the coelomocytes. By analyzing heavy metal and oxidative stress sensitivities of the coelomocyte-deficient *C. elegans* strain we discovered that coelomocytes are essential mainly for detoxification of heavy metals, but not of oxidative stress, a by-product of heavy metal toxicity.

**Conclusions/Significance:**

We established that HMT-1 from the multicellular organism confers tolerance to multiple heavy metals and is expressed in liver-like cells, the coelomocytes, as well as head neurons and intestinal cells, which are cell types that are affected by heavy metal poisoning in humans. We also showed that coelomocytes are involved in detoxification of heavy metals. Therefore, the HMT-1-dependent detoxification pathway and coelomocytes of *C. elegans* emerge as novel models for studies of heavy metal-promoted diseases.

## Introduction

Heavy metals are metallic elements with densities exceeding 5 g/cm^3^. Some heavy metals (*e.g.* copper [Cu], zinc [Zn] and iron [Fe]) at low concentrations serve as micronutrients, but are toxic when in excess [1], [2]. Nonessential heavy metals and metalloids (*e.g.* cadmium [Cd], arsenic [As] and mercury [Hg]) are toxic even at low concentrations [2]–[4]. The chronic exposure of humans to heavy metals either from occupational hazard, or from food and air, leads to their accumulation in tissues and causes various diseases, including neurodegenerative conditions, dysfunction of vital organs, and cancer [2]–[6]. Therefore, developing methods for protecting and detoxifying organisms in metal-polluted environments is contingent on our understanding of the effective cellular detoxification mechanisms.

In this regard, half-molecule ATP-binding cassette (ABC) transporters of the HMT-1 subfamily (**h**eavy **m**etal **t**olerance factor 1) and **p**hyto**c**helatin (PC) synthases, (γ-glutamylcysteinyltransferases; EC 2.3.2.15), are acutely required for detoxification of Cd [7]–[14]. It was suggested that HMTs act after PCSs by sequestering Cd coordinated to products of PCS-1 activity, phytochelatins (PC) into the vacuole, a lysosomal-like compartment of plant and fungi cells [15]. Our genetic studies in *C. elegans*, however, have shown that *pcs-1* and *hmt-1* do not act in a linear metal detoxification pathway [8]. These observations were further supported by our biochemical studies in *S. pombe* showing that SpHMT1 is not a primary Cd·PC transporter, and that an HMT-1 from *Drosophila*, an organism that lacks PC synthase homologs in its genome, is involved in Cd detoxification [16]. Furthermore, Preveral *et al*., also observed that SpHMT1 confers Cd tolerance in a PC-independent manner [17]. Interestingly, of heavy metals tested, SpHMT1 conferred tolerance only to Cd, but not to As, Cu, Hg, Ag and Sb [16], [17].

Here, we tested if HMT-1 from the multicellular organism, *C. elegans*, would confer tolerance only to Cd, or unlike SpHMT1, to other heavy metals as well. Since *C. elegans* has highly differentiated muscular, nervous, digestive and reproductive systems, and yet is comprised of only 959 optically transparent somatic cells, we determined the spatial distribution of *hmt-1* expression and related it to the expression pattern of its homolog in humans.

We established that unlike SpHMT1, CeHMT-1 conferred tolerance to As and Cu in addition to Cd. Consistent with the notion that *pcs-1* and *hmt-1* operate in distinct metal detoxification pathways, double *pcs-1;hmt-1* mutant worms were more sensitive to these metals than single *pcs-1* or *hmt-1* knock-out worms. In addition, *hmt-1* and *pcs-1* were expressed in distinct tissues, but co-expressed in coelomocytes, which are cells that are distributed in the pseudocoelom (body cavity) of *C. elegans* [18]. The latter finding was intriguing because the biological role of coelomocytes in *C. elegans* is not known [18]. However, because coelomocytes actively and continuously endocytose fluid and macromolecules from the pseudocoelom and because the pseudocoelomic fluid serves as the circulatory system for nutrients that are secreted into the pseudocoelom by the intestinal cells, it has been suggested that coelomocytes may act as liver cells by reprocessing and detoxifying pseudocoelomic fluid from harmful ingested substances [18], [19]. Since *pcs-1* and *hmt-1* function in detoxification of heavy metals and are also co-expressed in coelomocytes, we hypothesized that coelomocytes may be needed for heavy metal detoxification. By testing heavy metal sensitivity of a coelomocyte-deficient, NP717, strain, we uncovered the essential role of these cells in the detoxification of Cd, As and Cu. Since the coelomocytes deficiency did not increase the sensitivity of worms to hydrogen peroxide, we concluded that these cell types detoxify heavy metals, but not the oxidative stress, a consequence of many stresses as well as heavy metal toxicity. We also showed that although *hmt-1* may function in coelomocytes, it acts mainly outside these cell types in metal detoxification.

Given that the HMT-1 counterparts in mammals, HsABCB6 and RnABCB6, are involved in homeostasis of the essential heavy metal Fe or confer tolerance to Cu, respectively, and that HsABCB6 is expressed in similar tissues in humans as HMT-1 in *C. elegans* [20]–[22], the HMT-1-dependent metal detoxification pathway of *C. elegans* emerges as a novel model for studies of heavy metal-related diseases, such as neurodegenerative conditions similar to Parkinson's disease, dysfunction of digestive tract, and cancer [1], [2], [5], [6].

## Results and Discussion

### Analyses of *hmt-1* Deletion Alleles of *C. elegans*

Previous reverse genetic studies of *hmt-1* and *pcs-1* in *C. elegans* relied on expression knockdown by RNAi [7], [8]. Here, we used *hmt-1* deletion alleles, *gk155* and *gk161*, and *pcs-1(tm1748)* allele. At the onset of our studies, we determined the position of deletions by sequencing gDNAs isolated from *hmt-1(gk155)*, *hmt-1(gk161)* and *pcs-1(tm1748)* worms.

**Figure 1:**
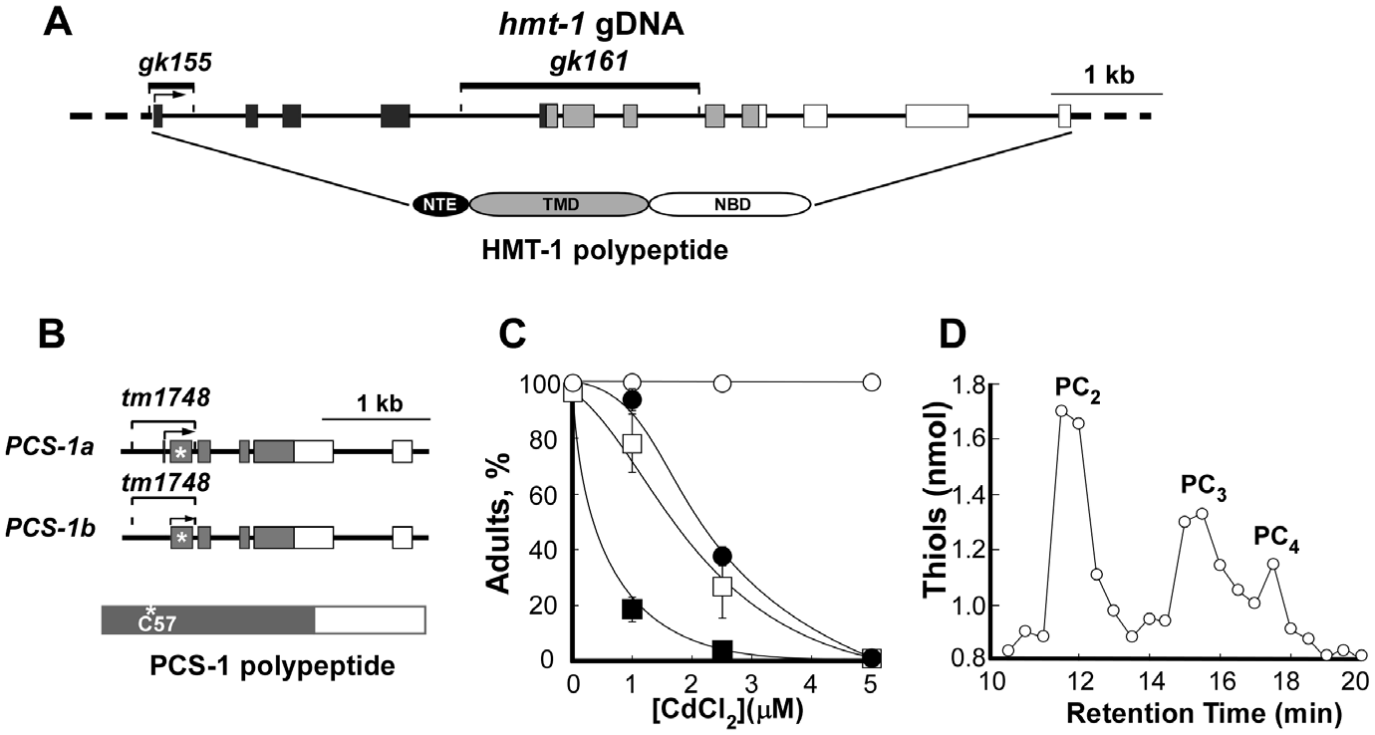
Molecular and functional analyses of *hmt-1* and *pcs-1* deletion alleles. **A.** Structure of the *hmt-1* gene, location of deletion mutations and domains of CeHMT-1 polypeptide. The black boxes depict regions encoding the N-terminal extension (NTE), light gray boxes depict regions encoding the transmembrane domain (TMD), and white boxes depict regions encoding the nucleotide-binding domain (NBD). The positions of *gk155* and *gk161* deletions are represented as lines above the genomic structure. **B.** Structure of the *pcs-1* gene, location of the deletion mutation and domains of the PCS-1 polypeptide. The grey boxes represent regions that encode a conserved N-terminal part of PCS-1. An asterisk indicates a catalytic Cys^57^ residue of the conserved catalytic triad of the PCS-1 polypeptide. PCS-1a and PCS-1b are predicted splice variants of PCS-1. In **A** and **B**, the exons and the introns are depicted as boxes and as connecting lines, respectively. **C.** Cd hypersensitivity of *hmt-1* and *pcs-1* deletion mutants. Wild-type N2 (○), *hmt-1(gk155)* (•), *hmt-1(gk161* (□) and *pcs-1(tm1748)* (▪) adult hermaphrodites were placed individually onto NGM plates with indicated concentrations of Cd and allowed to lay eggs. Shown are the percentages of the progeny that had reached adulthood 4 days after hatching. The total number of worms used for each strain and condition, and statistical significance of measurements are shown in Table S1. **D.** Reverse-phase HPLC analysis of phytochelatins in lysates prepared from Cd-grown N2 wild-type worms. The peaks designated “PC_2_”, “PC_3_” and “PC_4_” were identified on the basis of their co-migration with the *in vitro* synthesized PC standards [41].

Sequencing analysis revealed that *hmt-1*(*gk155)* has a 416 bp deletion, encompassing the 1^st^ exon and part of the 1^st^ intron (Figure 1A). An in-frame start codon is present downstream of the deletion breakpoint, at the beginning of the 2^nd^ exon. RT-PCR analysis disclosed the presence of a truncated transcript (data not shown), therefore, a truncated, *M_r_* 87,900, polypeptide lacking two cytosolic loops and a transmembrane domain of the NTE (N-terminal extension) could be generated in *hmt-1(gk155)* (Figure 1A). Nevertheless, given that the NTE is necessary for the activity of some full-molecule ABC transporters, we suspect that even if a truncated polypeptide were generated in this mutant allele, it would not be functional [23], [24]. Consistent with this suggestion, we established that although *hmt-1(gk155)* worms were indistinguishable from the wild type worms in a medium devoid of heavy metal, they were hypersensitive to Cd (Figure. 1C).

Allele *gk161* is a 2149 bp deletion of the 5^th^, 6^th^ and 7^th^ exons (Figure 1A). PCR analysis using oligos that are specific for the internal part of the deletion verified that the deleted region is absent (data not shown). Sequencing analysis established that the transcriptional fusion of the 4^th^ and 8^th^ exons introduced a premature stop codon. Therefore, if a translated truncated polypeptide (*M_r_* 22,800) were stable, it would lack the TMD and NBD. These domains, however, are essential for ABC transporters function [25]. Therefore, we suggested that *gk161* is a null allele of *hmt-1*. Consistent with this suggestion, *hmt-1(gk161)* worms were hypersensitive to Cd (Figure 1C; Table S1).

We did not find significant differences in the sensitivity of *gk155* and *gk161* alleles to Cd (Figure 1C; Table S1). Since the HMT-1 polypeptide of *gk161* allele lacked TMD and NBD that are required for the function of ABC transporters, we used this strain in our studies.

### Analyses of *pcs-1* Deletion Alleles of *C. elegans*

Sequencing analysis of gDNA from *pcs-1(tm1748)* worms revealed a 588 bp deletion and 3 bp insertion that have led to the removal of a part of the 5′ untranslated region and exons 1 and 2 of the predicted splice variant *PCS-1a*, or a part of 5′ untranslated region and exon 1 of the predicted splice variant *PCS-1b* (Figure 1B). An in-frame start codon is present downstream of the deletion breakpoint, at the beginning of the 3^rd^ exon in *PCS-1a* (or 2^nd^, in *PCS-1b*) that generated a stable transcript (data not shown). Nevertheless, if a truncated 35 kDa polypeptide in this allele were stable, it would not be functional since the deletion removed an essential Cys^57^ residue of the catalytic triad that is located in the conserved N-terminal part of PC synthases (Figure 1B, [26]–[28]). Consistent with this suggestion was finding that *pcs-1(tm1748)* worms were hypersensitive to Cd (Figure 1C; Table S1). Furthermore, a comparison of concentrations at which *pcs-1(tm1748)* and *hmt-1(gk161)* worms were able to reach adult stage after 4 days of culturing disclosed that *pcs-1(tm1748)* worms were much more sensitive to Cd than *hmt-1(gk161)* worms (Figure 1C; Table S1).

Finally, *pcs-1(tm1748)* worms failed to accumulate PC when cultured in medium supplemented with Cd. Reverse-phase HPLC analysis of non-protein thiols in lysates from Cd-cultured wild-type N2 worms revealed prominent peaks whose migration properties were indistinguishable from those of PC_2_, PC_3_ and PC_4_ standards (Figure 1D). The aggregate content of PC-thiols in N2 worms was 17 nmol/mg protein. In contrast, the content of PC-related thiols in lysates from Cd-cultured *pcs-1(tm1748)* worms was below the limit of detection (not shown).

Based on the position of the deletion, the acute hypersensitivity of *pcs-1(tm1748)* worms to Cd and their failure to synthesize PCs, we concluded that *tm1748* is a null allele of *pcs-1*.

### In Addition to Cd, CeHMT-1 Confers Tolerance to As and Cu

**Figure 2:**
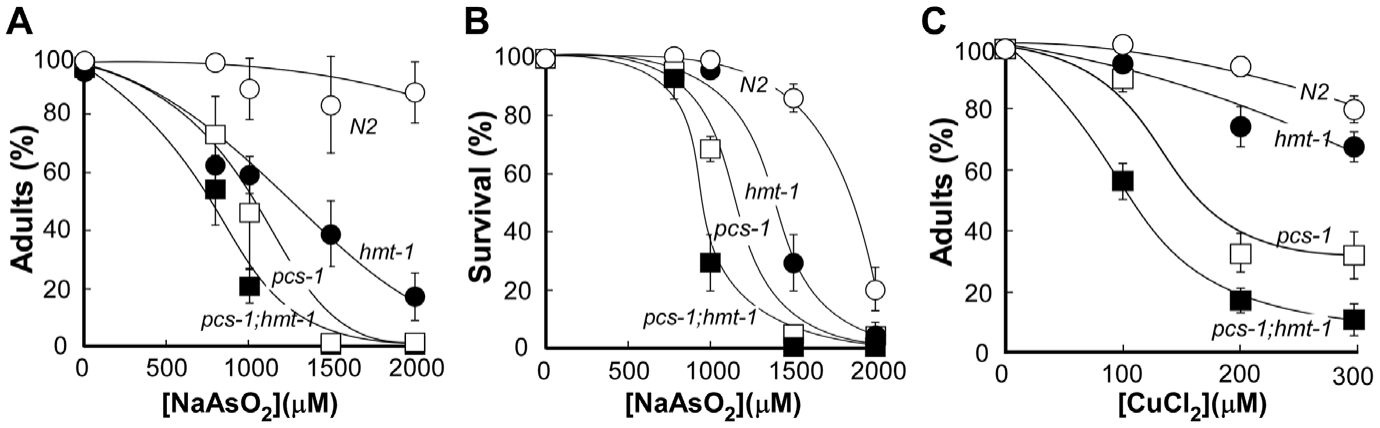
*hmt-1* is required for As and Cu tolerance and acts independently from *pcs-1*. Wild-type N2 (○, ***N2***), *hmt-1(gk161*) (•, ***hmt-1***), *pcs-1(1748)* (□, ***pcs-1***) and *pcs-1(tm1748);hmt-1(gk161)* (▪, ***pcs-1;hmt-1***) adult hermaphrodites were placed individually onto NGM plates with indicated concentrations of As (**A, B**) or Cu (**C**). As and Cu sensitivities were analyzed in the progeny after 4 days of culturing at 20°C. The percentages of worms that had reached adulthood 4 days after hatching are shown in **A** and **C**. The percentages of adult worms that had survived on medium with As after 5 days of culturing are shown in **B**. The total number of worms used for each strain and condition, and statistical significance of measurements are shown in Tables S2, S3.

Previous studies showed that SpHMT1 confers tolerance only to Cd [16], [17]. To determine if CeHMT-1 is involved in detoxification of other heavy metals, we tested the sensitivity of *hmt-1(gk161)* worms to As and Cu. In doing so we established that unlike SpHMT1, CeHMT-1 conferred tolerance to As and Cu in addition to Cd (Figure 2, Tables S2, S3). Indeed, *hmt-1(gk161)* worms were 1.6- and 5-fold more sensitive to the lowest (800 µM) and highest (2000 µM) concentrations of As than wild-type worms were (Figure 2A, Table S2).

Increasing concentrations of Cu, also affected the development of *hmt-1(gk161)* worms (Figure 2C, Table S3). At 200 µM of Cu, 92.4±2.76% of N2 worms reached the adult stage, whereas only 73.8±6.9% of *hmt-1(gk161)* worms were adults. At the highest concentration of Cu (300 µM), 80.5±4.35% of wild-type worms have reached the adult stage, whereas only 67.2±5.3% of *hmt-1(gk161)* worms were adults (Figure 2C, Table S3). Comparison between the percentage of *hmt1(gk161)* worms that have reached the adult stage in the medium with highest concentrations of As or Cu revealed that *hmt-1(gk161)* worms were 3.8-fold more sensitive to As than to Cu (Tables S2, S3).

It is noteworthy that the HMT-1 homolog from a mammal, RnABCB6, confers tolerance to Cu as well [22]. These data, along with our results showing that unlike SpHMT-1, CeHMT-1 confers tolerance to multiple heavy metals and that HMT1 of *S. pombe* groups separately from HMTs of *C. elegans* and mammals in the HMT-1 subcluster when their polypeptide sequences are subjected to phylogenetic analysis [16], suggest that the function of HMTs in multicellular organisms have diverged from SpHMT1.

### PCS-1 Is Also Required for Detoxification of Arsenic and Copper

PCS-1 of *C. elegans* confers Cd tolerance by catalyzing synthesis of heavy metal-binding Cys-rich peptides, phytochelatins (Figure 1D, [7], [29]). Since PCSs of *S. pombe* and *Arabidopsis* confer tolerance to multiple heavy metals [12]–[14], [30], we hypothesized that in addition to Cd, CePCS-1 would confer tolerance to other heavy metals too. As predicted, *pcs-1(tm1748)* worms were hypersensitive not only to Cd, but also to As and Cu (Figure 2A–C, Tables S2, S3).

Similar to Cd (Figure 1C), *pcs-1(tm1748)* mutant worms were more sensitive to As and Cu compared to *hmt-1(gk161)* mutants (Figure 2A, B, Tables S2, S3). Based on the percentage of worms that have reached the adult stage in the medium where heavy metals left N2 worms unaffected (As [1000 µM] or Cu [200 µM]), *pcs-1(tm1748)* worms were 1.3-fold more sensitive to As and 2-fold more sensitive to Cu than *hmt-1(gk161)* and 2- and 1.2-fold more sensitive than N2 worms.

### Arsenic Affects Viability of Worms

To determine whether chronic exposure to Cd, As or Cu affects the viability of worms that have reached adult stage after 4 days of culturing, we analyzed them after an additional 24 h (corresponding to 5 days of culturing in the presence of heavy metals). Chronic exposure of wild-type, *pcs-1(tm1748)* and *hmt-1(gk161)* mutants to Cd or Cu did not affect the viability of worms that have reached adult stage (not shown). However, the viability of adult worms in the medium with As was significantly affected (Figure 2B, Table S2). When the viability of worms was compared at the highest used concentration of As (2000 µM), *hmt-1(gk161)* and *pcs-1(tm1748)* worms were 3.8- and 4.5-fold more sensitive respectively than N2 worms.

### *pcs-1* and *hmt-1* Do Not Act in a Linear Pathway in Detoxification of As or Cu

We previously showed that *hmt-1* and *pcs-1* do not act in concert to detoxify Cd [8], [16]. To determine if they would share the same or distinct pathways in detoxifying As and Cu, we compared heavy metal sensitivity of single and double knockout animals. Our expectation was that if *hmt-1* acts in a distinct pathway in detoxifying As and/or Cu, its loss-of-function in conjunction with *pcs-1* may increase Cd hypersensitivity of *hmt-1* mutants (*i.e.* due to additive effects of two compromised detoxification pathways). On the other hand, if *hmt-1* acts in the same pathway as *pcs-1*, heavy metal hypersensitivity may not increase.

Our studies showed that a double *pcs-1(tm1748);hmt-1(gk161)* knockout was more sensitive to As and Cu than single *pcs-1(tm1748)* or *hmt-1(gk161)* knockouts regardless whether worms were scored as a number of individuals that have reached the adult stage, or as the number of adults that have died in the medium with As (Figure 2A–C). When scored as the percentage of worms that have reached the adult stage in the medium with the lowest concentration of As (800 µM), *pcs-1(tm1748);hmt-1(gk161)* worms were 1.2-, 1.4- and 1.8-fold more sensitive than *hmt-1(gk161), pcs-1(tm1748)*, or N2 worms respectively. When scored as the percentage of worms that have reached the adult stage when cultured in the medium with the lowest concentration of Cu (100 µM), *pcs-1(tm1748);hmt-1(gk161)* worms were 1.6-, 1.7-, and 1.8-fold more sensitive than *pcs-1(tm1748), hmt-1(gk161)* or N2 worms respectively.

These data are consistent with our previous genetic and biochemical studies of the HMT-1-dependent heavy metal detoxification pathway [8], [16] and show that *hmt-1* and *pcs-1* do not act in a linear pathway for the detoxification of other heavy metals as well as Cd.

### *hmt-1* and *pcs-1* Are Expressed in Distinct Cell Types of *C. elegans*, but Are Co-Expressed in Coelomocytes

To study the expression patterns of *hmt-1* and *pcs-1* and determine which tissues and cell types might be involved in the detoxification of heavy metals, we generated transgenic worms expressing GFP under the control of *hmt-1* or *pcs-1* promoters (*phmt-1::GFP* and *ppcs-1::GFP*, respectively). We chose the VF1.1 line from five independent transgenic lines exhibiting the same pattern of *phmt-1::GFP*-derived fluorescence and the VF15.1 line from nine independent transgenic lines exhibiting the same pattern of *ppcs-1::GFP*-derived fluorescence for subsequent studies.

**Figure 3:**
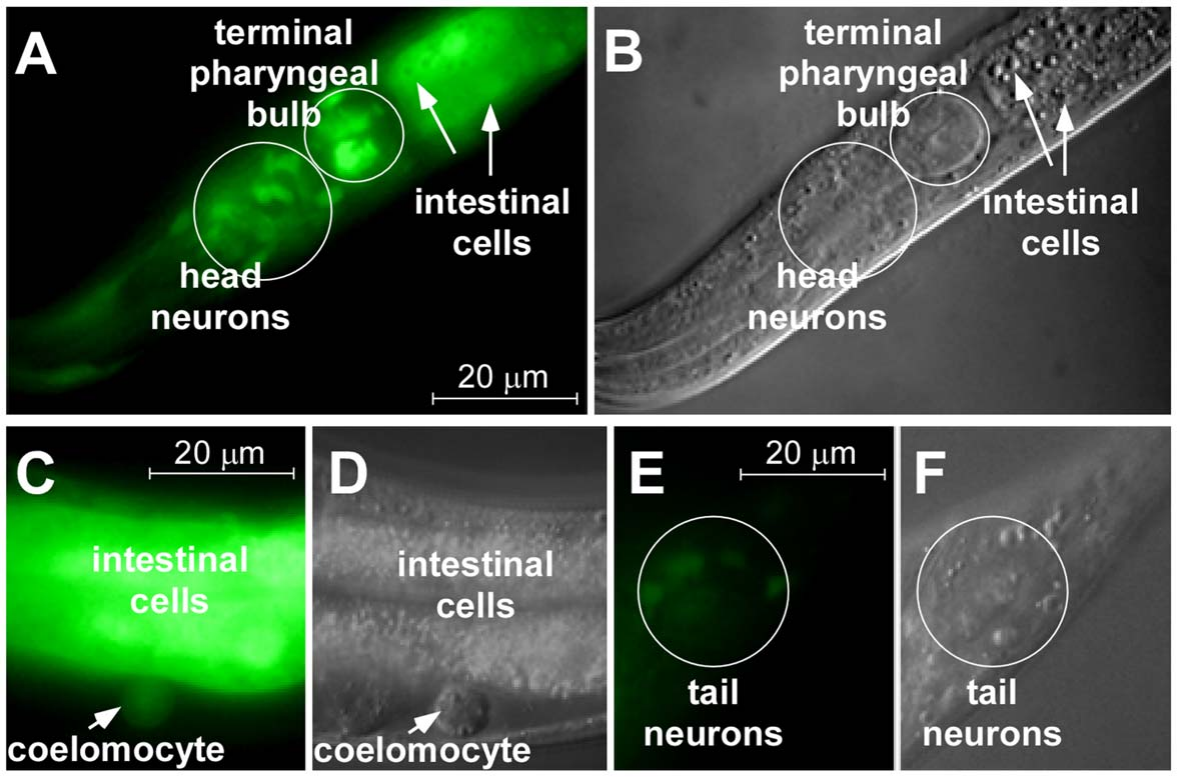
Fluorescence (A, C, E) and differential interference contrast (DIC [B, D, F]) microphotographs showing the expression pattern of *hmt-1*. Expression of *hmt-1* was analyzed by following the distribution of GFP-mediated fluorescence in transgenic animals expressing GFP from the *hmt-1* promoter. For visualizing neurons, we used worms at the L2 larval stage (**A, B**), while we used adults for visualizing GFP-mediated fluorescence in other tissues and cell types (**C, D, E, F**). Worms were immobilized in 20 mM NaN_3_. Epifluorescence micrographs were captured using the GFP-specific filter set and an AxioCam MRc camera interfaced with the Zeiss Axioscope 2 plus microscope. Tissues expressing *phmt-1::GFP* are indicated with arrows.

Analysis of the distribution of GFP-mediated fluorescence in the VF1.1 line disclosed the *hmt-1* promoter activity in intestinal cells and head and tail neurons (Figure 3). Expression of *hmt-1* in intestinal cells, but not in head and tail neurons was also observed by Zhao et al [31]. This discrepancy could be due to the absence of some regulatory elements in a construct that was used for generating transgenic animals in Zhao et al [31]. We recently confirmed the pattern of *hmt-1*-expression observed in our study using a rescuing *hmt-1* genomic fragment (*Kim, S., Sharma, A., Vatamaniuk, O.K. in preparation*).

**Figure 4:**
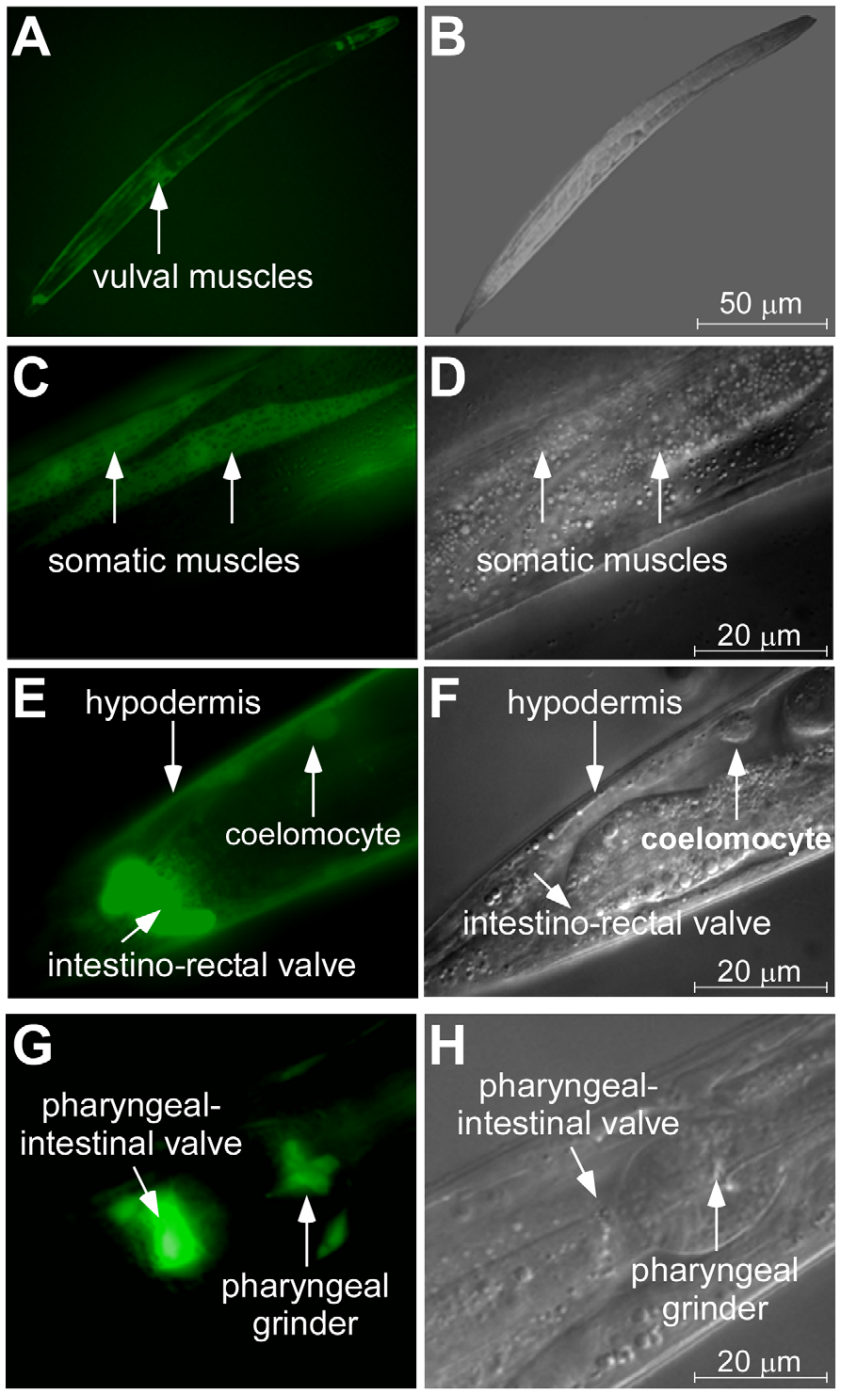
Fluorescence (A, C, E, G) and DIC (B, D, F, H) images showing the expression pattern of *pcs-1*. Transgenic adult worms expressing *GFP* from the *pcs-1* promoter were immobilized in 20 mM NaN_3_ and analyzed by DIC and epifluorescence microscopy. Cell types expressing *ppcs-1::GFP* are indicated with arrows.

Analysis of the distribution of the GFP-mediated fluorescence in the VF15.1 strain showed the *pcs-1* promoter activity in the hypodermis, the pharyngeal grinder, the pharyngeal-intestinal valve, and the bodywall and vulval muscles, but not in tissues and cell types expressing *hmt-1* (Figure 4). This observation is consistent with the suggestion that *pcs-1* and *hmt-1* act in distinct tissues and agrees with our genetic studies showing that *hmt-1* and *pcs-1* function in distinct detoxification pathways.

**Figure 5:**
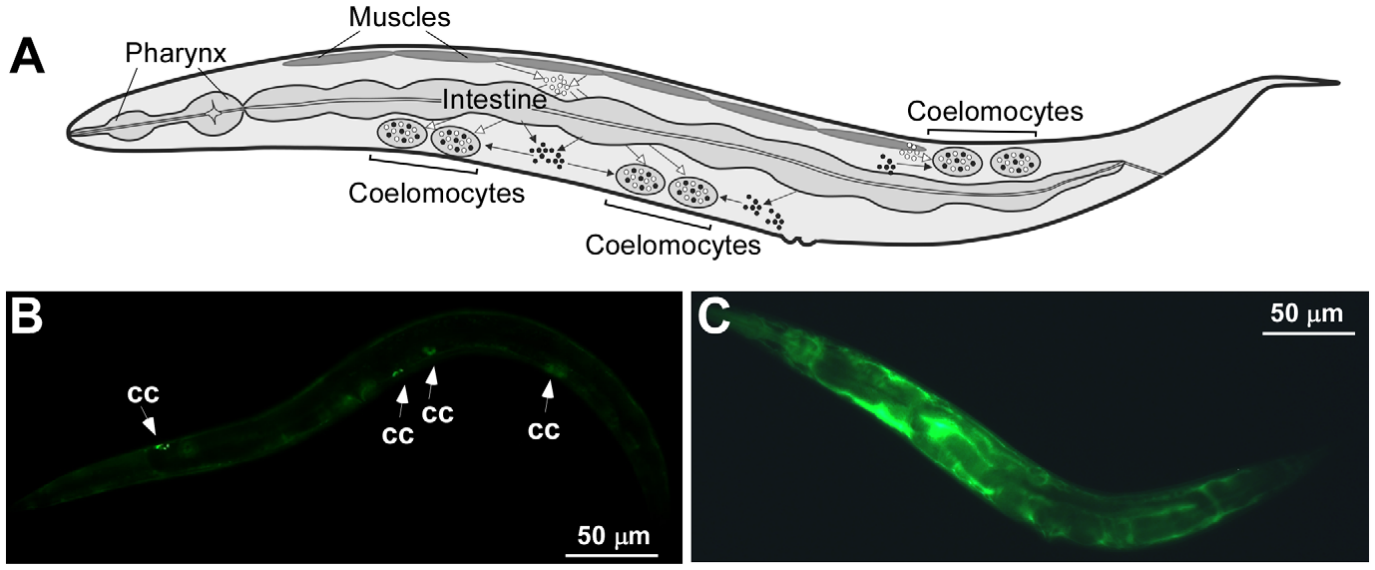
Visualizing the coelomocyte deficiency. A schematic drawing of a worm shows that fluids secreted into the pseudocoelom from surrounding tissues accumulate in coelomocytes (**A**, modified from Fares and Greenwald, 2001 [18]). Epifluorescence micrographs of GS1912 (**B**) and NP717 (**C**) worms. In GS1912, ssGFP is expressed in body wall muscles from *myo-3* promoter, secreted into the pseudocoelom and accumulated in coelomocytes [18]. White arrows indicate accumulation of GFP in coelomocytes (**cc, B**). As a result of coelomocytes ablation in NP717, GFP accumulates in the pseudocoelom [18] (**C**).

Although the bulk of the GFP expression, driven by *hmt-1* or *pcs-1* promoters was found in distinct tissues, we also detected GFP in coelomocytes of both, *phmt-1::GFP* and *ppcs-1::GFP* transgenic animals. Coelomocytes are large, ovoid mesodermal cells that are distributed as three pairs in the pseudocoelom (body cavity) and continuously and nonspecifically endocytosing pseudocoelomic fluid (Figure 5A, [18]). Coelomocytes have been regarded to function as a primitive liver, although toxin-mediated ablation of **coelomocytes** results in viable animals under standard laboratory conditions [18]. Therefore, the function of coelomocytes in *C. elegans* is still unknown.

### Coelomocyte-Deficient Worms Are Sensitive Mainly to Heavy Metals, but Not to Oxidative Stress

Finding that genes required for heavy metal detoxification, *hmt-1* and *pcs-1*, were expressed in coelomocytes raised the intriguing possibility that coelomocytes, whose function was unknown, might be involved in heavy metal detoxification. To test the function of coelomocytes in heavy metal detoxification, coelomocyte-deficient worms were assayed for sensitivity to Cd, Cu and As.

The coelomocyte-deficient strain, NP717, was generated by expressing a variant of the Diphtheria toxin A fragment (E148D) in worms under the control of the coelomocyte-specific *unc-122* promoter as described previously [18], [32], [33]. In addition, the NP717 strain was engineered to express ssGFP that is secreted into the pseudocoelom from bodywall muscles [18]. If coelomocytes are present and functional, the ssGFP is taken-up and degraded by coelomocytes as shown for strain GS1912 (Figure 5B, [18]). The lack of coelomocytes function in NP717 worms was confirmed by the accumulation of ssGFP in the pseudocoelom (Figure 5B, C, [18]).

**Figure 6:**
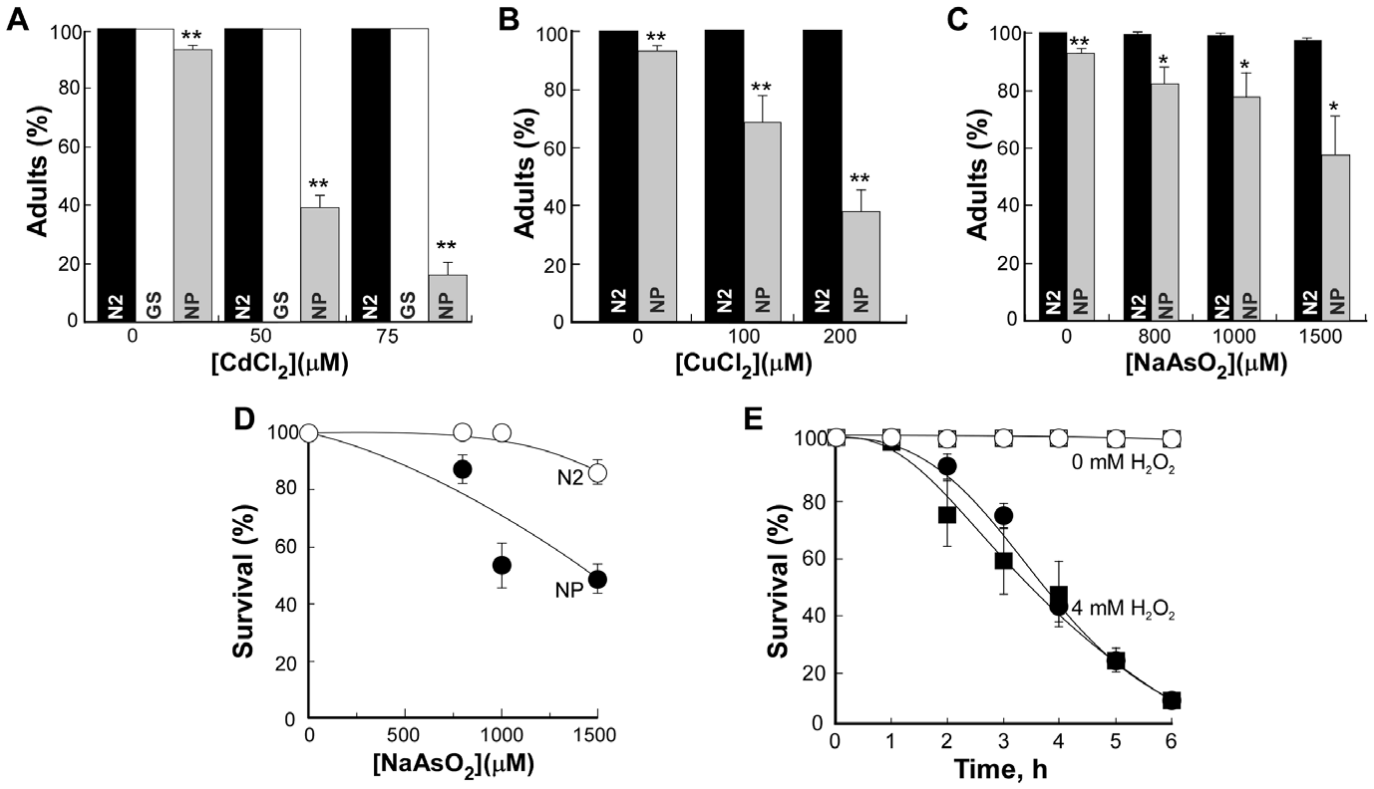
Coelomocyte-deficient worms are sensitive mainly to heavy metals, but not to oxidative stress. N2 wild-type (**N2**), GS1912 (**GS**) and NP717 (**NP**) adult hermaphrodites were placed onto NGM plates supplemented with indicated concentrations of CdCl_2_ (**A**), CuCl_2_ (**B**) or NaAsO_2_ (**C, D**). The percentages of worms that had reached adulthood 4.5 days after hatching are shown in **A, B** and **C**. The percentages of adult worms that had survived on the medium with As after 5.5 days of culturing are shown in **D**. The asterisks indicate statistically significant differences (*p≤0.05, **p≤0.01). Numbers of worms analyzed for each strain and condition are presented in Table S4. **E.** N2 (○) or NP717 (□) adult worms were incubated on standard NGM plates (**0 mM H_2_O_2_**). N2 or NP717 worms, incubated on plates with 4 mM H_2_O_2_ (**4 mM H_2_O_2_**) are indicated as • and ▪ respectively. Sixty worms were tested for each condition and each stain. *p*-values are indicated in the main body of the manuscript.

Analysis of heavy metal sensitivity showed that the development of coelomocyte-deficient worms was prematurely arrested or delayed in medium supplemented with heavy metals (Figure 6, Table S4). At 50 µM of Cd, only 38.5±4.3% of NP717 worms had reached adult stage, whereas 100% of GS1912 and N2 control worms were egg-laying adults (Figure 6A, Table S4). At higher concentration of Cd (75 µM), only 15.9±4.6% of NP717 worms had reached adult stage, whereas 99.6±0.4% of GS1912 and 100% of N2 worms were young adults (Figure 6A, Table S4). We also observed a pronounced sensitivity of NP717 worms to Cu. The highest concentration of Cu used in this experiment (200 µM) allowed 100% of N2 wild-type worms to reach adulthood compared to 37.2±6.9% of NP717 worms (Figure 6B, Table S4). Increasing concentrations of As in the medium affected developmental rates and viability of NP717 worms as well (Figure 6C, D; Table S4).

To test if the observed Cd, As and Cu sensitivity of coelomocyte-deficient worms is specific to metals, we examined the response of NP717 worms to oxidative stress, an inevitable consequence of many stresses as well as heavy metal toxicity [2]. Response to oxidative stress was evaluated by comparing the ability of NP717 and wild-type worms to survive in the presence of a reactive oxygen species (ROS), hydrogen peroxide (H_2_O_2_). Results showed that the viability of NP717 worms was indistinguishable from wild-type worms when cultured on NGM plates lacking H_2_O_2_ (Figure 6E). Addition of H_2_O_2_ to the medium significantly affected the survival of N2 and NP717 worms (*p* = 0.016, and *p* = 0.005 respectively). Although the viability of NP717 worms in the presence of H_2_O_2_ appeared to be lower than of wild-type worms, this difference was not statistically significant (*p* = 0.829). Therefore, we concluded that coelomocytes are mainly involved in detoxification of heavy metals, but not of oxidative stress.

### *hmt-1* Acts Primarily outside Coelomocytes

To determine whether *hmt-1* acts *via* coelomocytes, we generated coelomocyte-deficient *hmt-1(gk161)* worms (VF14 strain) and compared their Cd sensitivity with sensitivities of *hmt-1(gk161)* worms and of coelomocyte-deficient, NP717, worms. If *hmt-1* acts in coelomocytes, their absence may not increase heavy metal sensitivity of *hmt-1(gk161)* worms. In contrast, if *hmt-1* does not act in coelomocytes, Cd sensitivity of coelomocyte-deficient *hmt-1(gk161)* worms might increase (due to an additive effect of a compromised *hmt-1-*dependent and coelomocyte-dependent detoxification pathway).

**Figure 7:**
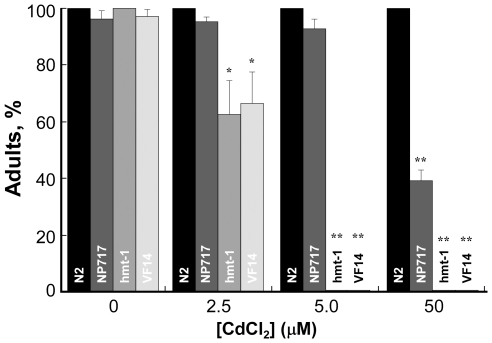
Cadmium sensitivity of coelomocyte-deficient *hmt-1(gk161)* mutant worms. Wild- type (**N2**), coelomocyte-deficient (**NP717**), *hmt-1(gk161)* (**hmt-1**), and coelomocyte-deficient *hmt-1(gk161)* (**VF14**) adult hermaphrodites were placed on the NGM medium supplemented with indicated concentrations of CdCl_2_. Worms were analyzed for heavy metal sensitivity after 4.5 days of culturing at 20°C. Shown are the percentages of worms that had reached adulthood 4.5 days after hatching. The asterisks indicate statistically significant differences (*p≤0.05, **p≤0.01). Numbers of worms analyzed for each strain and condition are presented in Table S5.

Comparison of Cd sensitivity of *hmt-1(gk161)*, coelomocyte-deficient, and coelomocyte-deficient *hmt-1(gk161)* worms yielded the following observations: 1) at heavy metal concentrations required for about 50% decrease in the number of worms that have reached adulthood, *hmt-1(gk161)* worms were about 20-fold more sensitive to Cd than coelomocyte-deficient worms (Figure 7, Table S5); 2) ablation of coelomocytes in *hmt-1(gk161)* worms did not increase Cd sensitivity of *hmt-1* mutants (Figure 7, Table S5). The latter observation is consistent with *hmt-1* acting, at least in part, *via* coelomocytes. Nevertheless, considering that *hmt-1(gk161)* worms with or without coelomocytes were much more sensitive to Cd than coelomocyte-deficient worms having functional *hmt-1*, we concluded that *hmt-1* acts mainly outside coelomocytes, but may act in part in coelomocytes. Our future analysis of the effect of the cell-type specific expression of HMT-1 in transgenic *hmt-1(gk161)* worms on their heavy metal sensitivity will identify tissues and cell types requiring HMT-1 for heavy metal detoxification.

### Concluding Remarks

Four central conclusions can be derived from these investigations. *First*, CeHMT-1 is distinct from its *S. pombe* counterpart in that it confers tolerance not only to Cd, but also to other heavy metals and metalloids, As and Cu. These data along with observations that RnABCB6 provides Cu tolerance [22] and our previous phylogenetic analysis showing that SpHMT1 clusters separately from HMTs from *C. elegans*, *Drosophila* and mammals [16] suggest that the functions of HMTs in multicellular organisms have diverged from their counterpart in the unicellular organism *S. pombe*. *Second*, results of the genetic analysis of the relationship between *hmt-1* and *pcs-1* and the expression patterns of *hmt-1* and *pcs-1* substantiate our previous observations that these genes do not act in a simple linear heavy metal detoxification pathway. *Third*, although coelomocytes have been regarded to act as a primitive liver, their role in *C. elegans* was unknown. The findings reported here represent the first demonstration of the function of coelomocytes of *C. elegans*: these cell types are essential for detoxification of heavy metals, but not of ROS, which are by products of multiple stresses as well as heavy metal toxicity. Whether in addition to heavy metals coelomocytes detoxify other toxins, and whether these toxic substances accumulate in coelomocytes, merits in-depth investigation. *Fourth*, we showed that *hmt-1* is expressed in coelomocytes, head neurons, and intestinal cells. Given that HMT-1 counterpart of humans, *ABCB6*, is expressed in similar tissues and cell types, and since these tissues are affected by heavy metals [1], [2], [20], [21], [34], [35], future studies of the *hmt-1* pathway in *C. elegans* may lead to the development of novel models for studies of heavy metal-caused neurodegenerative conditions and diseases of the digestive tract.

## Materials and Methods

### *C. elegans* Culture Conditions and Strains

*C. elegans* strains were maintained at 20°C on solid Nematode Growth medium (NGM) using the *E. coli* OP50 strain as a food source [36]. Heavy metals or H_2_O_2_ were added to the nematode growth medium (NGM) at the concentrations indicated and sensitivity tests were performed as described below.

We used the following *C. elegans* strains in our studies:

Bristol N2 (wild-type, and parent of all mutant strains)

DP38: *unc-119 (ed3)III*

VF1: *unc-119(ed3)III;gfEx1[phmt-1::GFP;unc-119(+)]*

VF2: *pcs-1(tm1748)II*

VF3: *hmt-1(gk161)III*

VF8: *hmt-1(gk155)III*

VF9: *pcs-1(tm1748)II;hmt-1(gk161)III*

VF14: *hmt-1(gk161)III;arls37;cdls32(pcc1::DT-A(E148D);unc-119-pmyo-2::GFP)*

VF15: *unc-119(ed3)III; gfEx2[ppcs-1::GFP; unc-119(+)]*

NP717: *unc-119(ed3); arls37; cdls32(pcc1::DT-A(E148D); unc-119(+) pmyo-2::GFP)*

GS1912: *dpy-20(e1282); arls37(pmyo-3::ssGFP)*. In the GS1912 strain 79 amino acids from SEL-1, including a signal sequence, are fused to GFP (ssGFP). ssGFP is expressed in body wall muscles from the *myo-3* promoter. The GFP that is secreted into the pseudocoelom is endocytosed and is accumulated in coelomocytes [18].

Prior to analyses, *pcs-1(tm1748)*, *hmt-1(gk161)* and *hmt-1(gk155)* alleles were backcrossed six times to N2 using standard genetic techniques. Deletions were confirmed by PCR and gel electrophoresis. The boundaries of deletions were determined by sequencing gDNA isolated from mutant worms. The following primer pairs were used for PCR and sequencing:

*hmt-1(gk155):*

5′-CCACGATCCACGTAATTTAG

3′-CAGACATTTCCGCTTTCAAC

*hmt-1(gk161):*

5′-TGAGCGGTGTGTAGAGTTGG

3′-TTCACTGGCTTTTGCCTTCT

*pcs-1(tm1748):*

5′- TTTCGAATGGCCACGCTATG

3′- CATGCCGACTCGAGCTGTTA

*pcs-1* and *hmt-1* are located on chromosome II and III respectively. To create double mutants *hmt-1(gk161)* was mated to *pcs-1(tm1748)* using standard genetic techniques [36]. The presence of *hmt-1(gk161)* and *pcs-1(tm1748)* deletions in the generated VF9 *pcs-1 (tm1748)II; hmt-1(gk161)III* strain was confirmed by PCR and gel electrophoresis.

### Generation of the Coelomocyte-Deficient Worms

The coelomocyte deficiency of worms (NP717 strain) was achieved by toxin-ablation due to the expression the Diphtheria toxin A fragment (E148D), which possesses 1.6% of the wild-type activity, under the control of a coelomocyte-specific promoter [18], [32]. Specifically, pJF143 plasmid (expressing the Diphtheria toxin A fragment bearing an E148D substitution replacing GFP in plasmid pcc1) and pHD137 plasmid (expressing both wild-type *unc-119* and GFP in pharyngeal cells) were introduced into the strain NP660 [*unc-119(ed3); arIs37(pmyo-3::ssGFP)*] by ballistic transformation [18], [33], [37].

To ablate coelomocytes in *hmt-1(gk161)* worms, *hmt-1(gk161)* males were crossed to NP717 hermaphrodites. Coelomocyte-deficient *hmt-1(gk161)* worms (VF14 strain) were selected in F2 based on acute Cd sensitivity resulting from *hmt-1* deficiency (Figure 1C, [8] and accumulation of GFP in the pseudocoelom, resulting from the coelomocytes deficiency [18].

### Heavy Metal and Oxidative Stress Sensitivities Tests

For analysis of heavy metal sensitivity, adult worms (P0) were placed individually on NGM plates with or without heavy metals (two adults/plate) and allowed to lay eggs for 4–5 h at 20°C before the adult worms were removed and laid eggs were counted. The effect of heavy metals on hatching was analyzed after 24 h. Heavy metals in concentrations used did not appear to affect hatching of wild-type or mutant strains. Instead, heavy metal sensitivity of mutant strains was manifested as larval arrest or delay in larval stages. Therefore, we evaluated heavy metal sensitivity by assessing the percentage of worms from the total number of hatched worms that have reached the adult stage in media with heavy metals. We assessed heavy metal sensitivity after 4.0 or 4.5 days (for NP717 and VF14) of culturing, when hatched worms have reached the adult stage in control (without heavy metals) conditions. We evaluated the viability of worms after additional 24 h of culturing in the presence of heavy metals. Worms were considered dead if they would not move on a plate and would not respond to a gentle touch with a worm pick. Since we started these assays with synchronous population (eggs), all N2 worms and all worms of some of mutant strains were adults after 4 days of culturing in control (without heavy metals) conditions.

The sensitivity of NP717 to oxidative stress was tested by evaluating the viability of worms during culturing in the presence of hydrogen peroxide (H_2_O_2_). Freshly-made NGM plates with or without 4 mM H_2_O_2_ were seeded with *E. coli* OP50. After bacterial loan has dried-out, young adults of NP717 or N2 wild-type worms were placed on plates (10 worms/plate). The viability of worms was evaluated every hour. Worms were considered dead if they would not move on a plate and would not respond to a gentle touch with a worm pick.

The results represent mean values of at least three independent experiments each of which had three experimental replicates. Statistical significance of measurements was determined using ANOVA Single Factor Analysis. The total number of worms used in each experiment and condition is indicated in Tables S1–S5. The absence of standard error (S.E.) bars in some parts of some figures indicates that either all of worms had reached the adult stage or survived at the particular condition, or that the S.E. values were very low and thus S.E. bars were behind symbols.

### Measurement of PC Content in Worms

Adult hermaphrodites were collected from NGM plates with M9 medium and washed free from *E.coli* OP50 by three rounds of centrifugation (3,500×*g* for 2 min) and resuspension in M9 medium. Collected worms were inoculated at a concentration of 30 worms/100 µl into 250 ml of liquid S-medium with Fe-HBED as the Fe source and *E. coli* OP50 as the food source. Worms were cultured for 5 days before CdCl_2_ was added to a final concentration of 100 µM for the activation of PC synthesis. After 24 h of incubation in the presence of Cd, worms were collected, washed free from bacteria in S-buffer and resuspended in lysis buffer containing 50 mM TRIS-HCl, pH 7.8, 10 mM 2-mercaptoethanol and 1 mM phenylmethylsulfonyl fluoride (PMSF), and 1 µg/ml each of leupeptin, aprotinin, and pepstatin. Worms were broken by sonication at 4°C in lysis buffer and worm debris was cleared by centrifugation at 3,500×*g* for 10 min. PCs were analyzed in the supernatant by reverse-phase high-performance liquid chromatography (RP-HPLC) as described [7].

### Generation of Transgenic Worms Expressing *phmt-1::GFP*

To generate the transcriptional reporter, *phmt-1::GFP*, a 2.1 kb region of the genomic sequence immediately upstream of the start of the *hmt-1* coding sequence was PCR-amplified using 5′-CCCAG2GGGCCGCGGAAACTAGTTTTTTAAATTAATAAATT and 3′-AAGCCCATGGTACCGGATTTTTTGGCCTGAAAATCTATAA primer pairs, designed to introduce *Sac*II and *Kpn*I restriction enzyme recognition sites at the 5′ and 3′ends respectively. After restriction digestion, the PCR product was fused with the *gfp* gene of the pPD117.01 vector [38]. The resulting *pPD117.01-phmt-1::GFP* construct was co-injected at 80 ng/µl with the selectable marker, a plasmid carrying a functional *unc-119* gene (*unc-119(+)*, 100 ng/µl) into the gonadal syncytium of severely paralyzed (uncoordinated, Unc) *unc-119(ed3)* adult hermaphrodites [39], [40]. Non-unc transgenic animals exhibiting GFP-mediated fluorescence were selected using Leica MZ16FA automated fluorescence stereozoom microscope with Leica EL6000 metal halide illuminator. One line, VF1.1, of five independently-derived transgenic lines showing the same GFP expression pattern, was used for subsequent analyses.

### Generation of *ppcs-1::GFP* Expressing Transgenic Worms

The transcriptional reporter *ppcs-1::GFP* was constructed by placing the PCR-amplified 1589 bp genomic DNA fragment upstream of 5′ of the start of *pcs-1* into *Sph*I/*Kpn*I sites of the pPD117.01 vector [38]. The primer pairs for PCR-amplification of the *pcs-1* promoter were: 5′-CTCCAGAAGCATGCCTATTGTCCTGGGTGCGATATTCT and 3′- CCGACATGGTACCTTTTGAAGTGTCTGCAATTAT. The resulting *pPD117.01-ppcs1::GFP* construct (80 ng/µl) was co-injected with a plasmid, carrying a functional *unc-119* (100 ng/µl) into the gonadal syncytium of *unc-119 (ed3)* animals [39], [40]. Non-unc transgenic animals exhibiting GFP-mediated fluorescence were selected using Leica MZ16FA automated fluorescence stereozoom microscope equipped with Leica EL6000 metal halide illuminator. Nine independent transgenic lines exhibited a similar pattern of GFP-mediated fluorescence. One line, VF15.1, was used for subsequent analyses.

### Microscopy

Worms were mounted onto 2% agarose pads, immobilized in 20 mM NaN_3_ and viewed with a Zeiss Axioscope 2 plus microscope equipped with differential interference contrast (DIC), polarization, and fluorescence optics. As determined by comparing GFP-mediated fluorescence in mobile worms and NaN_3_-immobilized worms, this anesthetic did not affect the expression pattern of GFP. Micrographs were captured using a Zeiss AxioCam MRc camera and Zeiss AxioVision 4.6 software.

## Supporting Information

Table S1 Cadmium sensitivity of *pcs-1* and *hmt-1* knockout worms. Two adult hermaphrodites were placed per NGM plate with the indicated concentration of Cd and allowed to lay eggs for 4–5 h at 20°C before the adult worms were removed. Shown are the percentages of the progeny that had reached adulthood 4 days after hatching. Statistically significant difference between the mean values of N2 wild-type and mutant strains (p≤0.01) is indicated as *. Statistically significant difference between the mean values of *pcs-1(tm1748)* and each of an *hmt-1* knockout allele (p≤0.01) is indicated by the section sign.
(0.04 MB DOC)

Table S2 Arsenic sensitivity of different knockout alleles. Adult hermaphrodites were placed on NGM plate with the indicated concentration of As and allowed to lay eggs for 4–5 h at 20°C before the adult worms were removed. Shown are the percentages of the progeny that had reached adulthood 4 days after hatching. The viability of worms was evaluated after additional 24 h of culturing in the presence of As. The number of worms analyzed at different concentrations of As was: N2: 0 µM - 435; 800 µM - 362; 1000 µM - 222; 1500 µM - 128; 2000 µM - 146; *pcs-1(tm1748)*: 0 µM - 433; 800 µM - 142; 1000 µM - 98; 1500 µM - 96; 2000 µM - 91; *hmt-1(gk161)*: 0 µM - 392; 800 µM - 209; 1000 µM - 312; 1500 µM - 100; 2000 µM - 98; *pcs-1(tm1748); hmt-1(gk161)*: 0 µM - 382; 800 µM - 152; 1000 µM - 189; 1500 µM - 101; 2000 µM - 98. Statistically significant difference between the mean values of N2 and mutant strains (p≤0.05) is indicated as *. Statistically significant difference between the mean values of double *pcs-1(tm1748);hmt-1(gk161)* mutants and each of a *pcs-1* or *hmt-1* knockout allele (p≤0.05) is indicated by the section sign.
(0.04 MB DOC)

Table S3 Copper sensitivity of *pcs-1* and *hmt-1* knockout alleles. Two adult hermaphrodites were placed per NGM plate with the indicated concentration of Cu and allowed to lay eggs for 4–5 h at 20°C, before the adult worms were removed. Shown are the percentages of the progeny that had reached adulthood 4 days after hatching. The number of worms analyzed at different concentrations of CuCl_2_ was as follows: N2: 0 µM - 204; 100 µM - 155; 200 µM - 144; 300 µM - 152; *pcs-1(tm1748)*: 0 µM - 222; 100 µM - 120; 200 µM - 178; 300 µM - 96; *hmt-1(gk161)*: 0 µM - 135; 100 µM - 109; 200 µM - 95; 300 µM - 86; *pcs-1(tm1748);hmt-1(gk161)*: 0 µM - 153; 100 µM - 165; 200 µM - 83; 300 µM - 104. Statistically significant difference between the mean values of N2 wild-type and mutant strains (p≤0.05) is indicated as *. Statistically significant difference between the mean values of *pcs-1(tm1748)* and *hmt-1* or *pcs-1;hmt-1* knockout worms (p≤0.05) is indicated by the section sign. Statistically significant difference between the mean values of *hmt-1(gk161)* allele and *pcs-1(tm1748)* or *pcs-1;hmt-1* knockout alleles (p≤0.05) is indicated as ¶.
(0.04 MB DOC)

Table S4 Heavy metal sensitivity coelomocyte-deficient worms (NP717 strain). Two adult hermaphrodites from each strain were placed per NGM plate with the indicated concentration of heavy metal and allowed to lay eggs for 4–5 h at 20°C. Shown are the percentages of the progeny that had reached adulthood 4.5 days after hatching. Statistically significant difference between the mean values of N2 wild-type and mutant strains is indicated as * (p≤0.05) or ** (p≤0.01).
(0.04 MB DOC)

Table S5 Cadmium sensitivity of *hmt-1(gk161)* (VF3 strain), coelomocyte-deficient worms (NP717 strain) and coelomocyte-deficient *hmt-1(gk161)* worms (VF14 strain). Two adult hermaphrodites from each strain were placed per each NGM plate with the indicated concentration of Cd and allowed to lay eggs for 4–5 h at 20°C before the adult worms were removed. Shown are the percentages of the progeny that had reached adulthood 5 days after hatching. Statistically significant difference between the mean values of N2 wild-type and mutant strains is indicated as * (p≤0.05) or ** (p≤0.01).
(0.04 MB DOC)
