# Supplementary material for: Detoxification of Multiple Heavy Metals by a Half-Molecule ABC Transporter, HMT-1, and Coelomocytes of Caenorhabditis elegans
Source: PLoS One. 2010 Mar 5;5(3):e9564. doi: 10.1371/journal.pone.0009564 (PMC2832763; doi:10.1371/journal.pone.0009564)
Supplement: Table S1 — Cadmium sensitivity of pcs-1 and hmt-1 knockout worms. Two adult hermaphrodites were placed per NGM plate with the indicated concentration of Cd and allowed to lay eggs for 4–5 h at 20°C before the adult worms were removed. Shown are the percentages of the progeny that had reached adulthood 4 days after hatching. Statistically significant difference between the mean values of N2 wild-type and mutant strains (p≤0.01) is indicated as *. Statistically significant difference between the mean values of pcs-1(tm1748) and each of an hmt-1 knockout allele (p≤0.01) is indicated by the section sign. (0.04 MB DOC) [file pone.0009564.s001.doc]

**Table S1. Cadmium sensitivity of *pcs-1* and *hmt-1* knockout worms.**

| **Strains** | **0 µM CdCl_2_** | | **1 µM CdCl_2_** | | **2.5 µM CdCl_2_** | | **5 µM CdCl_2_** | |
| --- | --- | --- | --- | --- | --- | --- | --- | --- |
|  | **Adults (%); Mean + S.E.** | **Number of analyzed worms** | **Adults (%); Mean + S.E.** | **Number of analyzed worms** | **Adults (%); Mean + S.E.** | **Number of analyzed worms** | **Adults (%); Mean + S.E.** | **Number of analyzed worms** |
| ***N2*** | 100 | 350 | 100 | 196 | 100 | 312 | 100 | 165 |
| ***hmt-1(gk155)*** | 100 | 211 | 94.0 ± 2.1*^§^ | 110 | 37.5 ± 11.8*^§^ | 95 | 0* | 161 |
| ***hmt-1(gk161)*** | 96.2 ± 1.2 | 333 | 78.2±10.5*^§^ | 188 | 27.2 ± 3.2*^§^ | 194 | 0.3 ± 0.3* | 173 |
| ***pcs-1(tm1748)*** | 96.4 ± 1.4 | 210 | 18.7 ± 4.1*^§^ | 163 | 3.8 ± 2.5*^§^ | 132 | 0* | 133 |

Two adult hermaphrodites were placed per NGM plate with the indicated concentration of Cd and allowed to lay eggs for 4-5 h at 20°C before the adult worms were removed. Shown are the percentages of the progeny that had reached adulthood 4 days after hatching. Statistically significant difference between the mean values of N2 wild-type and mutant strains (*p* ≤ 0.01) is indicated as *. Statistically significant difference between the mean values of *pcs-1(tm1748)* and each of an *hmt-1* knockout allele (*p* ≤ 0.01) is indicated as ^§^.
