# Supplementary material for: Detoxification of Multiple Heavy Metals by a Half-Molecule ABC Transporter, HMT-1, and Coelomocytes of Caenorhabditis elegans
Source: PLoS One. 2010 Mar 5;5(3):e9564. doi: 10.1371/journal.pone.0009564 (PMC2832763; doi:10.1371/journal.pone.0009564)
Supplement: Table S2 — Arsenic sensitivity of different knockout alleles. Adult hermaphrodites were placed on NGM plate with the indicated concentration of As and allowed to lay eggs for 4–5 h at 20°C before the adult worms were removed. Shown are the percentages of the progeny that had reached adulthood 4 days after hatching. The viability of worms was evaluated after additional 24 h of culturing in the presence of As. The number of worms analyzed at different concentrations of As was: N2: 0 µM - 435; 800 µM - 362; 1000 µM - 222; 1500 µM - 128; 2000 µM - 146; pcs-1(tm1748): 0 µM - 433; 800 µM - 142; 1000 µM - 98; 1500 µM - 96; 2000 µM - 91; hmt-1(gk161): 0 µM - 392; 800 µM - 209; 1000 µM - 312; 1500 µM - 100; 2000 µM - 98; pcs-1(tm1748); hmt-1(gk161): 0 µM - 382; 800 µM - 152; 1000 µM - 189; 1500 µM - 101; 2000 µM - 98. Statistically significant difference between the mean values of N2 and mutant strains (p≤0.05) is indicated as *. Statistically significant difference between the mean values of double pcs-1(tm1748);hmt-1(gk161) mutants and each of a pcs-1 or hmt-1 knockout allele (p≤0.05) is indicated by the section sign. (0.04 MB DOC) [file pone.0009564.s002.doc]

**Table S2. Arsenic sensitivity of different knockout alleles.**

| **Strains** | **Adults (%); Mean + S.E.** | | | | |
| --- | --- | --- | --- | --- | --- |
|  | **0 µM NaAsO_2_** | **800 µM NaAsO_2_** | **1000 µM NaAsO_2_** | **1500 µM NaAsO_2_** | **2000 µM NaAsO_2_** |
| ***N2*** | 100 | 99.6 ± 0.3 | 88.3 ± 10.6 | 83.0 ± 17 | 87.5 ± 12.5 |
| ***pcs-1(tm1748)*** | 100 | 74.3 ± 12.1* | 46.5 ± 19.0* | 0 * | 0* |
| ***hmt-1(gk161)*** | 100 | 62.7 ± 2.8* | 60.0 ± 7.0*^§^ | 39.7±12.1*^§^ | 17.5 ± 7.5*^§^ |
| ***pcs1(tm1748);hmt-1(gk161)*** | 100 | 54.0 ± 15.3* | 21.0 ± 6.0*^§^ | 0*^§^ | 0*^§^ |
| **Strains** | **Viability (%); Mean + S.E.** | | | | |
| ***N2*** | 100 | 99.1 ± 0.88 | 97.9 ± 1.4 | 86.3 ± 3.8* | 20.2 ± 7.6 |
| ***pcs-1(tm1748)*** | 100 | 93.9 ± 5.1 | 68.5 ± 10.4*^§^ | 4.6 ± 2.4* | 4.5 ± 0.5* |
| ***hmt-1(gk161)*** | 100 | 97.1 ± 2.0 | 96.2 ± 2.2^§^ | 28.9 ± 9.8*^§^ | 5.5 ± 3.5* |
| ***pcs1(tm1748);hmt-1(gk161)*** | 100 | 91.2 ± 3.7 | 29.4 ± 9.8*^§^ | 3.3 ± 1.5*^§^ | 0* |

Adult hermaphrodites were placed on NGM plate with the indicated concentration of As and allowed to lay eggs for 4-5 h at 20°C before the adult worms were removed. Shown are the percentages of the progeny that had reached adulthood 4 days after hatching. The viability of worms was evaluated after additional 24 h of culturing in the presence of As. The number of worms analyzed at different concentrations of As was: **N2:** 0 μM – 435; 800 μM – 362; 1000 μM – 222; 1500 μM – 128; 2000 μM – 146; ***pcs-1(tm1748)*:** 0 μM – 433; 800 μM – 142; 1000 μM – 98; 1500 μM – 96; 2000 μM – 91; ***hmt-1(gk161)*:** 0 μM – 392; 800 μM – 209; 1000 μM – 312; 1500 μM – 100; 2000 μM – 98; ***pcs-1(tm1748); hmt-1(gk161)*:** 0 μM – 382; 800 μM – 152; 1000 μM – 189; 1500 μM – 101; 2000 μM – 98. Statistically significant difference between the mean values of N2 and mutant strains (*p* ≤ 0.05) is indicated as *. Statistically significant difference between the mean values of double *pcs-1(tm1748);hmt-1(gk161)* mutants and each of a *pcs-1* or *hmt-1* knockout allele (*p* ≤ 0.05) is indicated as ^§^.
