# Supplementary material for: Detoxification of Multiple Heavy Metals by a Half-Molecule ABC Transporter, HMT-1, and Coelomocytes of Caenorhabditis elegans
Source: PLoS One. 2010 Mar 5;5(3):e9564. doi: 10.1371/journal.pone.0009564 (PMC2832763; doi:10.1371/journal.pone.0009564)
Supplement: Table S3 — Copper sensitivity of pcs-1 and hmt-1 knockout alleles. Two adult hermaphrodites were placed per NGM plate with the indicated concentration of Cu and allowed to lay eggs for 4–5 h at 20°C, before the adult worms were removed. Shown are the percentages of the progeny that had reached adulthood 4 days after hatching. The number of worms analyzed at different concentrations of CuCl2 was as follows: N2: 0 µM - 204; 100 µM - 155; 200 µM - 144; 300 µM - 152; pcs-1(tm1748): 0 µM - 222; 100 µM - 120; 200 µM - 178; 300 µM - 96; hmt-1(gk161): 0 µM - 135; 100 µM - 109; 200 µM - 95; 300 µM - 86; pcs-1(tm1748);hmt-1(gk161): 0 µM - 153; 100 µM - 165; 200 µM - 83; 300 µM - 104. Statistically significant difference between the mean values of N2 wild-type and mutant strains (p≤0.05) is indicated as *. Statistically significant difference between the mean values of pcs-1(tm1748) and hmt-1 or pcs-1;hmt-1 knockout worms (p≤0.05) is indicated by the section sign. Statistically significant difference between the mean values of hmt-1(gk161) allele and pcs-1(tm1748) or pcs-1;hmt-1 knockout alleles (p≤0.05) is indicated as ¶. (0.04 MB DOC) [file pone.0009564.s003.doc]

**Table S3. Copper sensitivity of *pcs-1* and *hmt-1* knockout alleles.**

| **Strains** | **Adults (%); Mean + S.E.** | | | |
| --- | --- | --- | --- | --- |
|  | **0 µM CuCl_2_** | **100 µM CuCl_2_** | **200 µM CuCl_2_** | **300 µM CuCl_2_** |
| ***N2*** | 100 | 100 | 92.4 ± 2.76 | 80.5 ± 4.35 |
| ***pcs-1(tm1748)*** | 100 | 89.3 ± 2.2*^§¶^ | 32.4 ± 6.6*^§¶^ | 31.4 ± 7.3*^§¶^ |
| ***hmt-1(gk161)*** | 100 | 93.8 ± 1.8*^¶^ | 73.8 ± 6.9*^§¶^ | 67.2 ± 5.3^§¶^ |
| ***pcs-1(tm1748);hmt-1(gk161)*** | 100 | 56.3 ± 5.4*^§¶^ | 17.7 ± 4.0*^§¶^ | 11.2 ± 4.6*^§¶^ |

Two adult hermaphrodites were placed per NGM plate with the indicated concentration of Cu and allowed to lay eggs for 4-5 h at 20°C, before the adult worms were removed. Shown are the percentages of the progeny that had reached adulthood 4 days after hatching. The number of worms analyzed at different concentrations of CuCl_2_ was as follows: **N2:** 0 μM – 204; 100 μM – 155; 200 μM – 144; 300 μM – 152; ***pcs-1(tm1748)*:** 0 μM – 222; 100 μM – 120; 200 μM – 178; 300 μM – 96; ***hmt-1(gk161)*:** 0 μM – 135; 100 μM – 109; 200 μM – 95; 300 μM – 86; ***pcs-1(tm1748);hmt-1(gk161)*:** 0 μM – 153; 100 μM – 165; 200 μM – 83; 300 μM – 104. Statistically significant difference between the mean values of N2 wild-type and mutant strains (*p* ≤ 0.05) is indicated as *. Statistically significant difference between the mean values of *pcs-1(tm1748)* and *hmt-1* or *pcs-1;hmt-1* knockout worms (*p* ≤ 0.05) is indicated as ^§^. Statistically significant difference between the mean values of *hmt-1(gk161)* allele and *pcs-1(tm1748)* or *pcs-1;hmt-1* knockout alleles (*p* ≤ 0.05) is indicated as ^¶^.
