# Supplementary material for: Detoxification of Multiple Heavy Metals by a Half-Molecule ABC Transporter, HMT-1, and Coelomocytes of Caenorhabditis elegans
Source: PLoS One. 2010 Mar 5;5(3):e9564. doi: 10.1371/journal.pone.0009564 (PMC2832763; doi:10.1371/journal.pone.0009564)
Supplement: Table S4 — Heavy metal sensitivity coelomocyte-deficient worms (NP717 strain). Two adult hermaphrodites from each strain were placed per NGM plate with the indicated concentration of heavy metal and allowed to lay eggs for 4–5 h at 20°C. Shown are the percentages of the progeny that had reached adulthood 4.5 days after hatching. Statistically significant difference between the mean values of N2 wild-type and mutant strains is indicated as * (p≤0.05) or ** (p≤0.01). (0.04 MB DOC) [file pone.0009564.s004.doc]

**Table S4. Heavy metal sensitivity coelomocyte-deficient worms (NP717 strain).**

| **Heavy metal concentration** | | **N2** | | **NP717** | | **GS1912** | | |
| --- | --- | --- | --- | --- | --- | --- | --- | --- |
|  |  | **Adults (%);**  **Mean + S.E.** | **Number of analyzed worms** | **Adults (%);**  **Mean + S.E.** | **Number of analyzed worms** | **Adults (%);**  **Mean + S.E.** | **Number of analyzed worms** | |
| **0 μM** | | 100 | 667 | 92.9 ± 1.6 | 601 | 100 | 404 | |
| **CdCl_2_** | **50 μM** | 100 | 451 | 38.5 ± 4.3** | 175 | 100 | 248 | |
|  | **75 μM** | 100 | 298 | 15.9 ± 4.6** | 179 | 99.6 ± 4.6 | 182 | |
| **CuCl_2_** | **100 μM** | 100 | 180 | 67.9 ± 8.7** | 79 | N/A | | N/A |
|  | **200 μM** | 100 | 233 | 37.2 ± 6.9** | 112 | N/A | | N/A |
| **NaAsO_2_** | **800 μM** | 97.8 ± 1.6 | 158 | 82.2 ± 5.5* | 110 | N/A | | N/A |
|  | **1000 μM** | 97.0 ± 1.4 | 242 | 77.3 ± 8.78 | 95 | N/A | | N/A |
|  | **1500 μM** | 96.8 ± 1.6 | 117 | 57.4 ± 12.8* | 86 | N/A | | N/A |

Two adult hermaphrodites from each strain were placed per NGM plate with the indicated concentration of heavy metal and allowed to lay eggs for 4-5 h at 20°C. Shown are the percentages of the progeny that had reached adulthood 4.5 days after hatching. Statistically significant difference between the mean values of N2 wild-type and mutant strains is indicated as * (*p* ≤ 0.05) or ** (*p* ≤ 0.01).
