# Supplementary material for: Detoxification of Multiple Heavy Metals by a Half-Molecule ABC Transporter, HMT-1, and Coelomocytes of Caenorhabditis elegans
Source: PLoS One. 2010 Mar 5;5(3):e9564. doi: 10.1371/journal.pone.0009564 (PMC2832763; doi:10.1371/journal.pone.0009564)
Supplement: Table S5 — Cadmium sensitivity of hmt-1(gk161) (VF3 strain), coelomocyte-deficient worms (NP717 strain) and coelomocyte-deficient hmt-1(gk161) worms (VF14 strain). Two adult hermaphrodites from each strain were placed per each NGM plate with the indicated concentration of Cd and allowed to lay eggs for 4–5 h at 20°C before the adult worms were removed. Shown are the percentages of the progeny that had reached adulthood 5 days after hatching. Statistically significant difference between the mean values of N2 wild-type and mutant strains is indicated as * (p≤0.05) or ** (p≤0.01). (0.04 MB DOC) [file pone.0009564.s005.doc]

**Table S5. Cadmium sensitivity of *hmt-1(gk161)* (VF3 strain), coelomocyte-deficient worms (NP717 strain) and coelomocyte-deficient *hmt-1(gk161)* worms (VF14 strain).**

| **Strains** | **0 µM CdCl_2_** | | **2.5 µM CdCl_2_** | | **5 µM CdCl_2_** | | **50 µM CdCl_2_** | |
| --- | --- | --- | --- | --- | --- | --- | --- | --- |
|  | **Adults (%); Mean + S.E.** | **Number of analyzed worms** | **Adults (%); Mean + S.E.** | **Number of analyzed worms** | **Adults (%); Mean + S.E.** | **Number of analyzed worms** | **Adults (%); Mean + S.E.** | **Number of analyzed worms** |
| ***N2*** | 100 | 61 | 100 | 76 | 100 | 57 | 100 | 407 |
| ***NP717*** | 95.8 ± 2.8 | 142 | 94.2 ± 2.8 | 175 | 92.3 ± 3.6 | 160 | 38.5 ± 4.3** | 175 |
| ***hmt-1(gk161)*** | 100 | 183 | 62.1 ± 12.0* | 197 | 0** | 67 | 0** | 112 |
| ***VF14*** | 96.4 ± 2.9 | 181 | 66.1 ± 11.1* | 250 | 0** | 177 | 0** | 115 |

Two adult hermaphrodites from each strain were placed per each NGM plate with the indicated concentration of Cd and allowed to lay eggs for 4-5 h at 20°C before the adult worms were removed. Shown are the percentages of the progeny that had reached adulthood 5 days after hatching. Statistically significant difference between the mean values of N2 wild-type and mutant strains is indicated as * (*p* ≤ 0.05) or ** (*p* ≤ 0.01).
